# 

**Published:** 1915-03

**Authors:** 


					content^ tr^
Intestinal Stasis.
By Sir William Arbuthnot
F.R.C.S. ...
On Toxic Polyneuritis of the Motor Type.
By J. Michell Clarke,
M.A., M.D., LL.D., F.R.C.P.
On the Pernasal Operation for Frontal Sinus Suppuration.
By P. Watson-Williams, M.D.Lond.
24
Drainage Continu par le Gaz Oxygene.
Par M. le docteur Renoirdre
39
An Outbreak of Institutional Dysentery due to the Y. Bacillus.
By R. H. Norgate, M.R.C.S., and I. Walker Hall, M.D.
44
Progress of the Medical Sciences :
Medicine.
By J. R. Charles, M.A., M.D., B.C.Cantab., F.R.C.P.
54
Surgery.
By C. A. Moore, M.B., M.S. Lond., F.R.C.S.
58
Diseases of Children.
By E. Cecil Williams, M.B., B.C.,
B.A. Cantab.
6i
Reviews of Books
63
Editorial Note
65
Therapeutic Notes..
68
Meetings of Societies
7i
Local Medical Notes   72

				

